# Iron limitation promotes metabolic cross-feeding between cheese ripening bacteria

**DOI:** 10.1093/ismejo/wrag100

**Published:** 2026-04-24

**Authors:** Rina Mekuli, Dominique Swennen, Jean-Michel Camadro, Sophie Landaud, Vincent Hervé

**Affiliations:** UMR SayFood, Université Paris-Saclay, INRAE, AgroParisTech, 91120 Palaiseau, France; UMR SayFood, Université Paris-Saclay, INRAE, AgroParisTech, 91120 Palaiseau, France; Institut Jacques Monod, Université Paris Cité, CNRS, 75013 Paris, France; UMR SayFood, Université Paris-Saclay, INRAE, AgroParisTech, 91120 Palaiseau, France; UMR SayFood, Université Paris-Saclay, INRAE, AgroParisTech, 91120 Palaiseau, France

**Keywords:** metal, abiotic stress, metallophores, polyamines, signaling

## Abstract

Iron is a limiting micronutrient in various environments, and its scarcity orchestrates microbial interactions across diverse ecosystems. The cheese surface, which is oxic, iron-limited, and a host of moderately complex ecosystems, can serve as a model system to study iron-mediated microbial interactions. In this work, we focused on two ripening bacteria isolated from cheese, *Hafnia alvei* and *Brevibacterium aurantiacum*. We combine growth measurements, transcriptomics, proteomics, and metabolomics to examine the role of iron in their interactions within a synthetic medium designed to mimic late cheese ripening conditions, using mono and coculture systems under iron limitation. Coculturing resulted in significant differences in the physiology of both strains, with a more notable effect on *H. alvei*. *H. alvei*, the only siderophore producer of the two, appeared to experience iron limitation in the coculture. This is partially attributed to sharing siderophores, and thus, iron, with *B. aurantiacum*. Multi-omics analysis points to several key exchanges. First, putrescine acts as a cross-fed metabolite, where *B. aurantiacum* synthesizes it and *H. alvei* uses it as an energy source. Next, we found evidence for the activity of quorum sensing and potential quorum quenching mechanisms, previously implicated in siderophore biosynthesis. Additionally, coculturing led to increased production of volatile sulfur compounds, contributing to positive organoleptic characteristics of cheese. Our model system reveals the modifications of C, N, S metabolisms in response to an abiotic stress and provides a framework to study such responses in numerous iron-limited ecosystems.

## Graphical Abstract

**Graphical Abstract:**
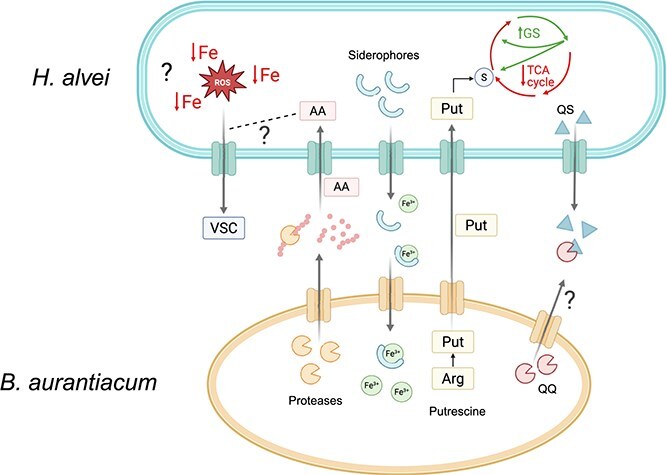

## Introduction

Iron, an essential micronutrient for most microorganisms, is involved in microbial metabolism at multiple levels, spanning from enzyme cofactors to gene regulation. It is incorporated in a substantial part of the proteome, most commonly through iron–sulfur ([Fe–S]) clusters, heme, or elemental form [1]. Its two main redox states, $\textnormal{Fe}^{\textnormal{2+}}$ and $\textnormal{Fe}^{\textnormal{3+}}$, enable it to both donate and accept electrons and, therefore, make it participatory in many reactions [2]. It is involved in aerobic and anaerobic respiration, DNA synthesis and repair, transcriptional and posttranscriptional modifications, oxidative stress sensing, and other anabolic and catabolic reactions. Iron is especially important in aerobic microorganisms, considering the extensive involvement of [Fe-S] clusters and iron-containing cytochromes in the electron transport chain (ETC), the primary source of ATP production in aerobic respiration [3–5]. Despite being an abundant mineral on Earth, its solubility remains low in many environments due to being in the ferric form under oxic conditions and at neutral pH. This low solubility restricts the availability of iron to microorganisms across diverse ecosystems, including marine environments, soils, and host–pathogen interfaces [6–9]. Similarly, the conditions present on the surface of cheese during the ripening process, characterized by both oxic conditions and a neutral pH, render the cheese matrix an iron-limited environment. In addition, the iron-chelating nature of milk proteins further contributes to a lower concentration of iron available to ripening microorganisms [10]. This required ripening bacteria to adopt sophisticated iron-scavenging and regulation mechanisms to combat iron limitation [11]. One of the iron acquisition strategies used under iron limitation is the production of iron-chelating molecules called siderophores. This strategy is broadly used by bacteria in various ecosystems [12].

Siderophores are structurally diverse molecules synthesized by microbes under iron-limited conditions to sequester iron from the environment [13]. However, not all microbes can synthesize them. Instead, if they have the corresponding transporters, auxotrophic bacteria depend on siderophores produced by other microbes [14]. Numerous cheese-associated microbes encode genes for siderophore transporters but not synthesis [11]. An analysis of 44 French Protected Designation of Origin cheeses, comprising 136 metagenomes and 1400 genomes and metagenome-assembled genomes (MAGs), revealed that iron/siderophore import-related pathways in MAGs are considerably more prevalent than siderophore biosynthesis ones [15]. This allows siderophores, and thus iron, to become one of the mediators of microbial interactions on the cheese surface [16–18]. Microbial communities are widespread across natural and synthetic ecosystems, and play essential roles in biogeochemical cycles, plant and animal health, biotechnological and medical innovations, as well as processing in the food industry [19]. Although these communities were long thought to be dominated by antagonistic interactions, cooperation also plays a major role in shaping their dynamics [20–22]. This behavior has been hypothesized by meta-transcriptomic analysis in mini-model cheeses, between *Hafnia alvei* and *Brevibacterium aurantiacum*, present in smear-ripened cheese [23]. These strains are important for organoleptic characteristics of cheese because they produce volatile sulfur compounds (VSCs) [24, 25]. VSCs have a low detection threshold and are major contributors to the typical aroma of cheese, especially in smear-ripened cheeses. In addition, *B. aurantiacum* also produces a distinct orange pigmentation on the surface of red-smear cheeses [26]. The harsh conditions on the cheese surface, being both iron and potentially oxygen-limiting, lead to difficulties in colonization for some bacteria. This is the case for *B. aurantiacum*, which is reported to be an obligate aerobe, and siderophore production is strain-dependent [27, 28]. In contrast, *H. alvei* is a facultative anaerobe and strains isolated from other environments have been shown to produce siderophores [29–31]. These characteristics make *H. alvei* more robust and *B. aurantiacum* more sensitive to the environment in cheese. However, *H. alvei* has been shown to have low proteolytic activity, and thus depends on other microbes to access nitrogen sources in cheese, such as *B. aurantiacum* [23].

This work will examine the interactions between *H. alvei* and *B. aurantiacum* in a synthetic medium designed to mimic the conditions present during the late stages of cheese ripening. We conducted experiments to investigate whether iron starvation induces metabolic changes in each strain, both individually and in coculture. We performed transcriptomic, proteomic, and metabolomic analyses, and assessed their physiological changes by comparing the omics profiles of the strains in coculture versus monoculture under iron deprivation.

## Materials and methods

### Strains and media

*Hafnia alvei* GB001 and *Brevibacterium aurantiacum* 8(6) were acquired from UMR0782 SayFood research unit (INRAE, Palaiseau, France). Both of these strains were originally isolated from cheese. They were grown in a synthetic medium formulated to mimic cheese ripening conditions, adapted from [24]. To achieve iron limitation in the medium, we used the iron chelator ethylenediaminedi-o-hydroxyphenylacetic acid (EDDHA) (BLD Pharmatech, Shanghai, China). The medium was composed of 25 g/l casein or casein hydrolysate (Merck, Darmstadt, Germany); 1.9 g/l yeast nitrogen base that does not contain any of the following: amino acids, ammonium, sulfate, iron, and copper (Formedium, Norfolk, England); 5 g/l glycerol; 5 g/l sodium lactate; 17 g/l sodium chloride; and 0.00004 g/l copper sulfate. It was supplemented with different levels of EDDHA, ranging from 0.5–5 $\mu $M EDDHA, solubilized in water with vigorous agitation, or 1.2 $\mu $M FeCl$_{3}$ to achieve the required iron concentrations. The pH was adjusted to 7. All glassware were rinsed with a HCl solution and low iron water to minimize iron contamination.

### Experimental design

An initial set of experiments was carried out to assess the growth of *H. alvei* and *B. aurantiacum* at different levels of iron availability. They were cultivated in monocultures using a cheese-like synthetic medium containing different levels of iron, via EDDHA or FeCl$_{3}$ supplementation. Both strains were first precultured in the medium with no addition of iron, in order to limit the amount of iron stored in their storage proteins. For *H. alvei*, we used casein hydrolysate as the nitrogen source, due to its low proteolytic activity on casein [23]. EDDHA concentrations of 5 $\mu $M, 2.5 $\mu $M, 1 $\mu $M, and 0.5 $\mu $M, as well as 1.2 $\mu $M of FeCl$_{3}$ were tested. Additionally, concentrations of 5 $\mu $M EDDHA and 1.2 $\mu $M FeCl$_{3}$ were included where casein was utilized as the nitrogen source for comparative reasons. For *B. aurantiacum*, casein was used as a nitrogen source because it is able to hydrolyze casein. Due to its higher sensitivity to iron limitation, *B. aurantiacum* was tested for a wider range of EDDHA concentrations: 5 $\mu $M, 2.5 $\mu $M, 2 $\mu $M, 1.5 $\mu $M, 1 $\mu $M, and 0.5 $\mu $M, as well as 1.2 $\mu $M FeCl$_{3}$. These experiments were carried out to select suitable levels of EDDHA and FeCl$_{3}$ to simulate an iron-restrictive and iron-sufficient environment.

For the preliminary experiments, cultures were grown in 250 ml Erlenmeyer flasks under agitation at 200 rpm at 25$^{\circ }$C. We monitored growth by measuring optical density at a wavelength of 600 nm. A chrome azurol S (CAS) assay for siderophore detection on agar plates was also performed, according to a previously described protocol [32], to assess siderophore synthesis. Levels of 5 $\mu $M EDDHA for *H. alvei* and 1.5 $\mu $M EDDHA for *B. aurantiacum* for the iron-limited condition were selected for further experiments.

The new set of experiments was conducted in similar conditions to the preliminary ones, but we used 2 l Erlenmeyer flasks for improved aeration. Oxygen availability was measured using noninvasive dissolved O$_{2}$ probes (PreSens Precision Sensing GmbH, Regensburg, Germany). Each strain was cultured in monoculture and in coculture at a 1:1 ratio of inoculation under iron limitation. To examine the inter-dependence of the strains, we used casein as a nitrogen source, and an EDDHA concentration of 5 $\mu $M for a more iron-limited environment. In these final experiments, we collected data on growth, dissolved oxygen, pH, as well as transcriptomic, proteomic, and metabolomic profiles at exponential growth under iron limitation. All experiments were performed in three biological replicates.

### Transcriptomic analysis

RNA extraction was done using the RNeasy Mini Kit from Qiagen (QIAGEN, Courtaboeuf, France), and was subsequently tested for quality using Agilent’s Tape Station (Agilent, Santa Clara, USA). rRNA depletion, library construction, and RNA sequencing were done by a service provider (QIAGEN Genomic Services, Hilden, Germany). QIAseq FastSelect Bacteria kit (QIAGEN) was used to reduce the amount of rRNA species. Library preparation was quality-controlled using capillary electrophoresis (Tape D1000). Based on the quality of the inserts and the concentration measurements, the libraries were pooled in equimolar ratios. The library pool(s) were quantified using qPCR. The library pools were then sequenced on a NextSeq 2000 System (Illumina) according to the manufacturer’s instructions. Raw data were de-multiplexed, and FASTQ files for each sample were generated using the bcl2fastq software v2.20.0.422 (Illumina).

The sequencing reads were trimmed in the presence of Illumina adapter sequences, and the quality was analyzed using Fastp 0.23.1 [33]; read sequences were mapped against the reference database (*B. aurantiacum* 8(6): NCBI Bioproject PRJEB19868, *H. alvei* GB001: NCBI Bioproject PRJEB6257) using STAR 2.7.11a [34]. Raw counts were extracted with featureCounts as part of the Subread 2.0.1 package [35]. The raw data were submitted to the NCBI repository with the accession number PRJNA1238254. Quality control of the RNAseq results was done with the SARTools pipeline [36].

The raw counts normalization and differential analysis results were obtained using the Bioconductor DESeq2 1.44.0 package [37]. Gene set enrichment was analyzed using the Bioconductor clusterProfiler 4.14.6 package [38].

The Snakemake 8.0.1 workflow [39] to obtain the raw counts matrix, the raw counts matrix, and the normalized counts matrix are available at the Recherche Data Gouv - Transcriptomic analysis repository.

### Proteomic analysis

Protein extraction was done using RIPA lysis and extraction buffer (Merck, Darmstadt, Germany) with Complete Protease Inhibitor Cocktail (Roche, Basel, Switzerland). The concentration was determined by BCA protein assay kit (Merck, Darmstadt, Germany), and it was confirmed by SDS-PAGE. The proteomic analysis was carried out by a service provider (Institut Jacques Monod, ProteoSeine, Paris, France). Samples were analyzed with a TimsTOF Pro 2 mass spectrometer (Bruker Daltonics, Bremen, Germany) coupled to an Evosep One system (Evosep, Odense, Denmark). The mass spectrometer was operated in Parallel Accumulation–Serial Fragmentation (PASEF) mode at a 1.3 second cycle time. Mass spectra for MS and MS/MS scans were recorded in the 100–1700 *m/z* range. Ion mobility was set to 0.75–1.25 V$\cdot $s/cm$^2$ over a ramp time of 180 ms. Data-dependent acquisition was done with 6 PASEF MS/MS scans per cycle with a near 100% duty cycle. Low *m/z* and singly charged ions were excluded through filtering in the *m/z* and ion mobility space. Dynamic exclusion was set to 0.8 min, with a target value of 16 000 and an intensity threshold of 1000. Collision energy was increased in steps as a function of ion mobility.

Raw files were processed using PEAKS Online 11 (build 1.9, Bioinformatics Solutions Inc.). The cutoff for identification at protein and peptide group levels was 1% false discovery rate. Label-free quantification was conducted using the PEAKS Online 11 quantification module. The analysis parameters included a mass error tolerance of 10 parts per million (ppm), a collision cross-section error tolerance of 0.02, and a retention time shift tolerance of 0.5 min for matching between runs. Protein abundance was determined using the top N peptide method, and total ion current for normalization. Multivariate statistics were performed using Qlucore Omics Explorer 3.9 (Qlucore AB, Lund, Sweden). For normalization, a positive threshold value of 1 was defined, ensuring all abundance values below this threshold were replaced with 1 before applying a log$_{2}$ transformation. The dataset is available at the Recherche Data Gouv - Proteomic analysis repository.

### Volatile metabolome analysis

The volatile organic compounds were detected using a dynamic headspace system (DHS) with a Gerstel MPS autosampler (Mülheim an der Ruhr, Germany) and gas chromatography–mass spectrometry analysis (GC-MS) with an Agilent 7890B GC system coupled to an Agilent 5977B quadrupole mass spectrometer (Agilent, Santa Clara, USA). Samples were incubated at 30$^{\circ }$C and agitated for 3 min. They were then purged by helium gas at 30 ml/min and concentrated in the Tenax trap maintained at 30$^{\circ }$C. This was followed by dry purging with helium gas at 50 ml/min at 30$^{\circ }$C for 6 min. A thermal desorption unit connected to a cooling injection system (TDU-CIS Gerstel) was used for desorption and cryofocusing. Starting with an initial temperature of 30$^{\circ }$C in the TDU, samples were heated to 290$^{\circ }$C at a rate of 60$^{\circ }$C/min, and then maintained at 290$^{\circ }$C for 7 min. The cryofocusing at CIS was done at −100$^{\circ }$C, followed by desorption at 270$^{\circ }$C at 12$^{\circ }$C/s. After, the compounds were injected in a polar capillary column (HP-Innowax, 60 m $\times $0.32 mm, 0.25 $\mu $m film thickness, PEG; Agilent, USA) carried by helium at a flow rate of 1.6 ml/min. The initial oven temperature was 40$^{\circ }$C for 5 min, followed by the first ramping up to 155$^{\circ }$C at 4$^{\circ }$C/min, andthen followed by the second ramping up to 250$^{\circ }$C at 20$^{\circ }$C/min. The temperature was then maintained at 250$^{\circ }$C for 5 min. The ionization in the quadrupole mass spectrometer was done in the electron-impact mode at 70 eV. The data were analyzed using the Agilent MassHunter Qualitative Analysis B.07.00 (Agilent, Santa Clara, USA), and peaks were annotated with the NIST 2017 Mass Spectral Library (Version 2.3). A standard mixture was injected every 10 injections, and samples were analyzed in a randomized sequence. Extraction reproducibility was assessed by comparing the peak areas of the standard injections, and a standard deviation of <10% was deemed acceptable. The annotated dataset is available at the Recherche Data Gouv - Metabolomic analysis repository.

### Data analysis and visualization

Data analysis and visualizations were performed in R software (version 4.1.2). Area Under Curve (AUC) values were extracted using Growthcurver (version 0.3.1) [40]. Ribosomal 30S and 50S subunits were selected using FetchMGs package (version 2.1.0). Gene set enrichment analysis visualization was conducted using the ClusterProfiler package (version 4.14.6). Other figures were generated with the ggplot2 package (version 3.5.1), using data analyzed as described in the transcriptomic, proteomic, and volatile metabolome analyses. Post hoc statistical analysis [Tukey’s Honestly Significant Difference (HSD)] for monoculture growth was conducted using the multcomp package (version 1.4.26). The significance of relative growth counts was assessed with the Wilcoxon rank-sum test from the R stats package (version 4.1.2). All experiments were performed in three biological replicates, and all statistical analyses were conducted on the corresponding replicates.

## Results

### *H. alvei* and *B. aurantiacum* exhibit lower growth in iron-limited conditions

**Figure 1:**
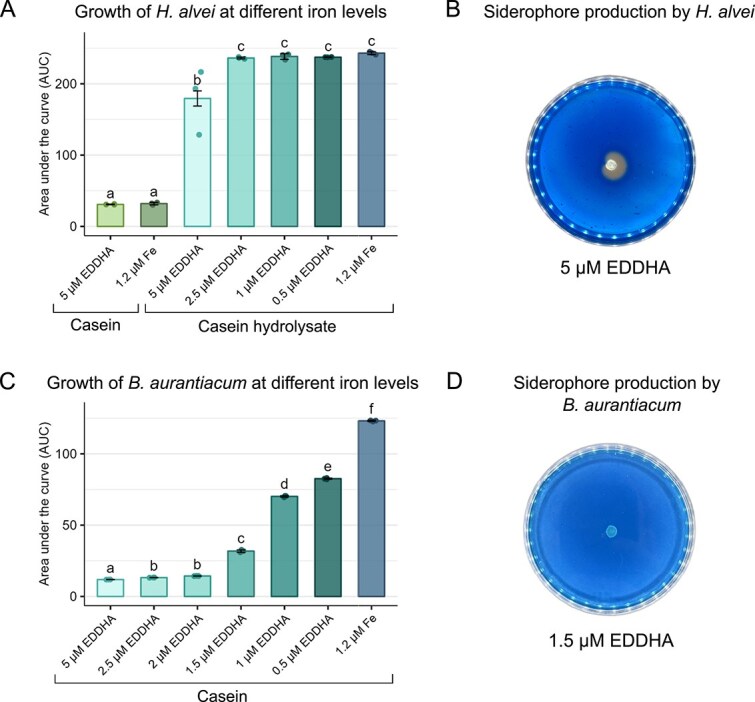
Growth of *H. alvei* and *B. aurantiacum* monocultures under different iron availabilities at 25$^{\circ }$C for 52 h. (A) AUC of *H. alvei* growth curves at different EDDHA and FeCl$_{3}$ concentrations, in casein and casein hydrolysate-based medium. (B) Siderophore production by *H. alvei* at 5 $\mu $M EDDHA as indicated by the presence of a halo in the CAS medium. (C) AUC of *B. aurantiacum* growth curves at different EDDHA and FeCl$_{3}$ concentrations, in casein-based medium. (D) The absence of siderophore production by *B. aurantiacum* at 1.5 $\mu $M EDDHA. Statistical analysis was performed using ANOVA followed by Tukey’s HSD.

To compare cellular changes imposed by low iron conditions, and the cross-feeding dynamics between *H. alvei* and *B. aurantiacum*, the analysis was conducted in monoculture and coculture, in iron-limited and iron-sufficient conditions. The preliminary growth experiments, conducted to select proper EDDHA concentrations to reach iron-scarcity, resulted in differences in growth compared with the iron-abundant condition (Fig. 1A and C).

For *H. alvei*, there was no significant effect in growth for EDDHA levels of 2.5 $\mu $M and lower, likely due to its ability to produce siderophores and efficiently scavenge iron (Fig. 1A). However, growth was significantly reduced at 5 $\mu $M EDDHA (*P* < 0.05); therefore, this concentration was selected for further analysis. We also carried out a CAS assay for siderophores, to ensure that *H. alvei* was producing siderophores at 5 $\mu $M EDDHA. The CAS test resulted positive for siderophore detection (Fig. 1B). In addition, culturing *H. alvei* in casein compared with casein hydrolysate resulted in poor growth, highlighting its dependence on the proteolytic activity of other microbes. In *B. aurantiacum*, the addition of EDDHA reduced growth in a dose-dependent manner, probably due to its inability to produce siderophores (Fig. 1C). We selected a concentration of 1.5 $\mu $M EDDHA for further analysis, where growth is significantly reduced (*P* < 0.05) but is sustained enough to acquire the relevant experimental data. Due to *B. aurantiacum* exhibiting proteolytic activity, all experiments were done in casein-based medium. In addition, the CAS assay resulted in a negative for siderophore detection (Fig. 1D).

Another set of experiments, now using the EDDHA concentrations selected in the preliminary experiments, was carried out. To investigate the metabolic changes occurring as a result of the two iron levels, we collected transcriptomic (Supplementary Table S1), proteomic (Supplementary Table S2), and volatile metabolomic data (Supplementary Table S3). Given the close link of iron metabolism to oxygen, we monitored dissolved oxygen levels throughout growth (Supplementary Fig. S1). This was done to ensure that the cells were not experiencing oxygen limitation at the time of sampling, thereby separating the effect of iron limitation from that of oxygen. We will now focus on the metabolic alterations observed when the bacteria are grown in coculture versus monoculture under low iron conditions, following the addition of EDDHA.

### Coculturing imposes a metabolic shift of central metabolism in *H. alvei*

**Figure 2:**
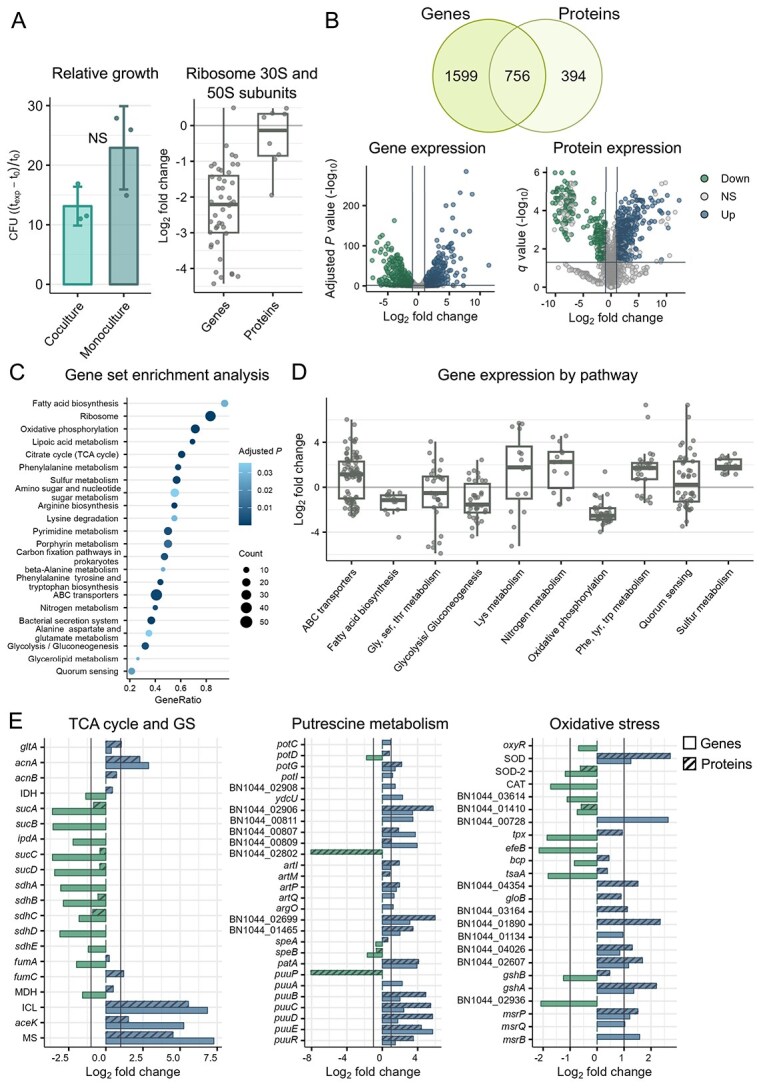
Differences across the growth, transcriptomic, proteomic, and metabolomic profile of *H. alvei* in coculture compared with monoculture. (A) Relative growth of *H. alvei* in coculture and monoculture (CFU ($t_{\textrm{exp}} - t_{0}$) / $t_{0}$), and expression of ribosomal genes and proteins (log$_{2}$ fold change). Statistical analysis was performed using the Wilcoxon Rank Sum Test: NS (not significant). (B) Significantly differentially expressed genes (DEGs) and proteins (adjusted *P* and *q* value < 0.05, unique peptides $\geq $ 3) and volcano plots of DEGs and proteins in coculture versus monoculture (adjusted *P* and *q* value < 0.05, unique peptides $\geq $ 3, log$_{2}$ fold change > 1). (C) Gene set enrichment analysis based on KEGG functional categories. (D) DEGs in relevant pathways (log$_{2}$ fold change). (E) Differential expression of genes and proteins in the TCA cycle and GS, putrescine metabolism, and oxidative stress (log$_{2}$ fold change).

Coculturing *H. alvei* with *B. aurantiacum* in low iron conditions resulted in an altered physiology of *H. alvei*. At the exponential phase, *H. alvei* exhibited lower growth, indicated by relative colony-forming unit counts (Fig. 2A). Consistently, most ribosomal 30S and 50S subunits were also downregulated in the coculture, both proteins and transcripts, indicating lower growth rates (Fig. 2A) [41]. Next, we analyzed the transcriptome and proteome of the coculture compared with the monoculture in the presence of EDDHA. Among the 2355 significantly expressed genes and 1150 proteins (adjusted *P* and *q* values < 0.05), 756 overlapped (based on UniProt accession numbers) (Fig. 2B). Gene expression analysis revealed 869 upregulated and 881 downregulated genes in the coculture (Fig. 2B). At the protein level, 490 proteins were upregulated, whereas 249 were downregulated (Fig. 2B). To identify the most affected pathways, we performed a gene set enrichment analysis based on KEGG categories (Fig. 2C). Twenty-two pathways were shown to be significantly enriched in the coculture compared with the monoculture, including glycolysis/gluconeogenesis, TCA cycle, oxidative phosphorylation, sulfur metabolism, metabolism of numerous amino acids, and quorum sensing (QS).

The majority of genes involved in glycolysis/gluconeogenesis, TCA cycle, and oxidative phosphorylation were downregulated (Fig. 2D and E). In contrast, the enzymes of the glyoxylate shunt (GS) were upregulated in the transcriptome and proteome (Fig. 2E) [42]. Consequently, the impaired function of the TCA cycle likely contributes to the downregulation of oxidative phosphorylation (Fig. 2D). These metabolic changes suggest a shift toward anaerobic respiration, further supported by the upregulation of formate dehydrogenase N and dimethyl sulfoxide reductase subunits (log$_{2}$ fold change > 1). Dissolved oxygen measurements did not indicate a lack of oxygen during growth, making the limitation of iron the underlying factor for this metabolic shift (see Supplementary Fig. S1).

Another upregulated pathway in *H. alvei* in the coculture is putrescine degradation. PatA, PatD, and the putrescine utilization (Puu) pathway are involved in putrescine degradation and utilization as a carbon source (Fig. 2E) [43, 44]. Putrescine/spermidine transporters were among the highest differentially expressed genes (DEGs) and proteins (Fig. 2E). Despite the upregulation of these transporters, arginine decarboxylase (gene) and agmatinase, which catalyze the conversion of arginine to agmatine and putrescine, were slightly downregulated (Fig. 2E), pointing to the import of these compounds.

#### Sulfur metabolism of *H. alvei* is induced in the presence of *B. aurantiacum*

**Figure 3:**
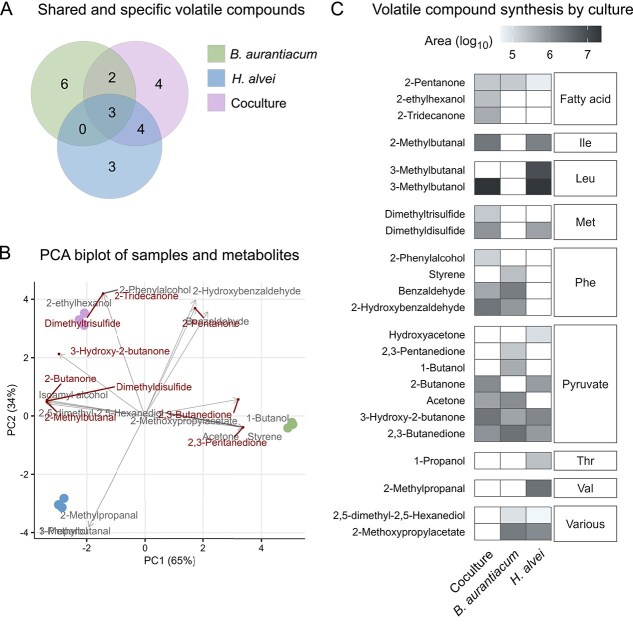
Profile of volatile metabolite synthesis for monocultures and coculture. (A) Shared and specific volatile compounds (*B. aurantiacum*  $n = $11, *H. alvei*  $n = $10, coculture $n = $13). (B) PCA biplot of samples and metabolites. Metabolites highly related to positive aromatic profile of cheese are denoted in red. (C) Synthesis of volatile compounds by each culture. White squares represent no synthesis. Abbreviations of precursors of metabolites: Ile, isoleucine; Leu, leucine; Met, methionine; Phe, Phenylalanine; Thr, threonine; Val, valine. See Supplementary Table S3 for standard deviation values.

A highly differentially expressed pathway in *H. alvei* is sulfur metabolism, which was induced in the coculture (Fig. 2D). Its upregulation could be linked to several factors. One factor could be oxidative stress. Glutathione, involved in protection from radical species, is a sulfur-containing molecule synthesized from cysteine and glutamate [45]. *H. alvei* seems to experience a certain level of oxidative stress. A superoxide dismutase, peroxidases, cysteine synthase, and glutamate–cysteine ligase were all upregulated (Fig. 2E). However the glutathione synthase gene, but not protein, and glutathione reductase were in fact downregulated, indicating the opposite (Fig. 2E). In addition, other genes and proteins indirectly related to oxidative stress, such as glutathione S-transferases which are more implicated in xenobiotic detoxification, and methionine sulfoxide reductases were also upregulated (Fig. 2E) [46, 47]. Another factor could be the higher availability of sulfur containing amino-acids in the coculture for *H. alvei*. Coculturing *H. alvei* with *B. aurantiacum* changed the expression levels of multiple ABC transporters (Fig. 2D). *H. alvei* was able to acquire amino acids in the coculture, likely from the proteolytic activity of *B. aurantiacum* on casein (upregulated proteases and peptidases in *B. aurantiacum* in coculture, see Supplementary Table S1). Degradation products of these amino acids were detected in the volatilome analysis, where some volatiles derived from leucine, isoleucine, and methionine precursors were present in higher quantities in the coculture (Fig. 3). The coculture yielded the greatest diversity of volatile compounds ($n = $13), including most of those responsible for desirable cheese aromas, such as methionine-derived sulfur volatiles (Fig. 3B).

### Coculturing *B. aurantiacum* with *H. alvei* alleviates the effect of iron limitation

**Figure 4:**
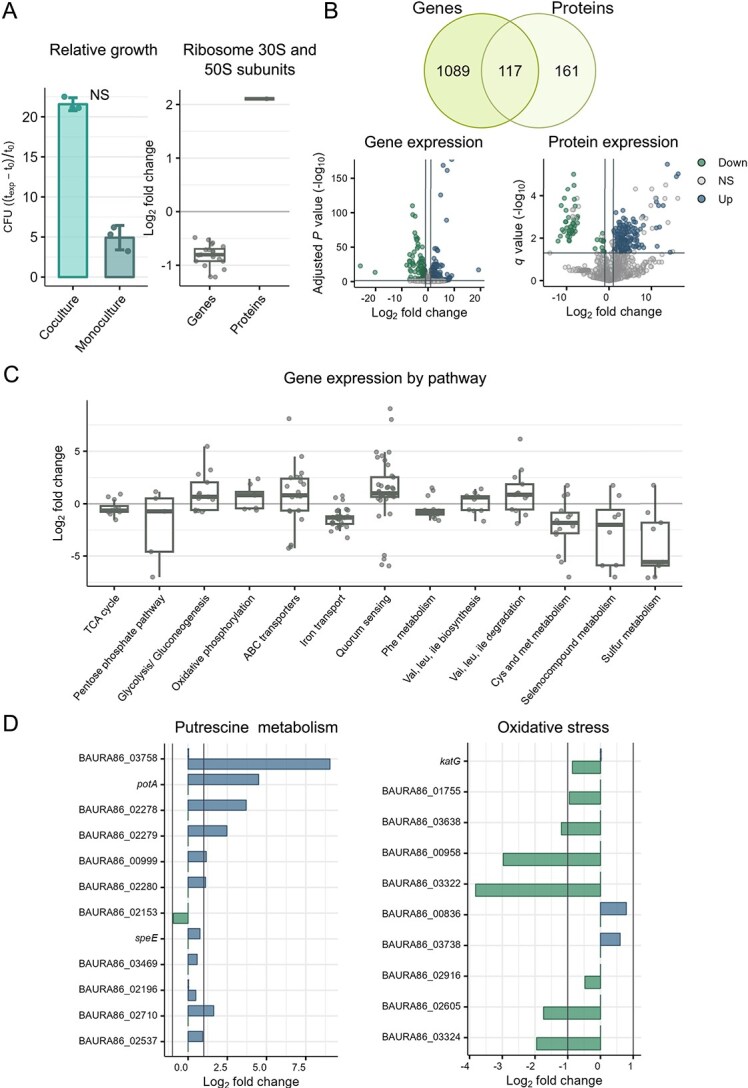
Differences across the growth, transcriptomic, proteomic, and metabolomic profile of *B. aurantiacum* in coculture compared with monoculture. (A) Relative growth of *B. aurantiacum* in coculture and monoculture (CFU ($t_{\textrm{ exp}} - t_{0}$) / $t_{0}$), and expression of ribosomal genes and proteins (log$_{2}$ fold change). Statistical analysis was performed using the Wilcoxon Rank Sum Test: NS (not significant). (B) Significantly DEGs and proteins (adjusted *P* and *q* value < 0.05, unique peptides $\geq $ 3) and volcano plots of DEGs and proteins in coculture versus monoculture (adjusted *P* and *q* value < 0.05, unique peptides $\geq $ 3, log$_{2}$ fold change > 1). (C) DEGs in relevant pathways (log$_{2}$ fold change). (D) Differential expression of genes in putrescine metabolism and oxidative stress (log$_{2}$ fold change).

Coculturing *B. aurantiacum* with *H. alvei* in iron-limited conditions resulted in a different response compared with *H. alvei*. Relative growth counts reveal that *B. aurantiacum* showed increased growth in the coculture (Fig. 4A). Ribosomal transcripts exhibited a slight decrease (log$_{2}$ fold change < 1), and only one ribosomal protein showed significant upregulation (Fig. 4A). In general, 1206 genes and 278 proteins were significantly expressed (Fig. 4B). The transcriptomic and proteomic analyses revealed only moderate changes between coculture and monoculture conditions compared with *H. alvei*. In the transcriptome, 313 genes were upregulated and 272 genes were downregulated, whereas in the proteome, 224 proteins were upregulated and 51 proteins were downregulated (Fig. 4B). Due to the low number of significantly expressed proteins, we will mostly focus on the transcriptomic analysis. The gene set enrichment analysis identified only one pathway as significantly enriched: iron transport. Consequently, we decided to analyze DEGs of pathways of interest.

Overall, there were few pathways with considerable differential expression (Fig. 4C). In contrast to *H. alvei*, genes in glycolysis/gluconeogenesis and oxidative phosphorylation were slightly upregulated in the coculture, hinting that *B. aurantiacum* was not experiencing iron limitation. Furthermore, most genes in the TCA cycle were not highly differentially expressed. In fact, only aconitase showed a decrease of more than twofold in coculture (−1.52 log$_{2}$ fold change). Aconitase is a bifunctional enzyme which may be upregulated in the absence of iron [48]. Similar to *H. alvei*, metabolism related to arginine, putrescine, and spermidine was generally upregulated in the coculture (Fig. 4D). We investigated the putrescine degradation pathway to assess if *B. aurantiacum* was using putrescine as a carbon source. Genes associated with putrescine utilization were either absent or not differentially expressed. However, putrescine/spermidine transporters were highly upregulated in the coculture (Fig. 4D). Key enzymes in arginine degradation to putrescine and spermidine, namely spermidine synthase, arginine decarboxylase, and agmatinase, are slightly upregulated (Fig. 4D). Agmatinase, which catalyzes the conversion of agmatine to putrescine, showed an increase in expression >2 folds (1.6 log$_{2}$ fold change). These findings indicate that although putrescine and spermidine are being synthesized, they are likely being exported rather than utilized as energy sources.

We observed in the coculture that iron transport genes are mostly downregulated (Fig. 4C). These genes encode iron complex transport systems and siderophore reductases.

A major difference between *B. aurantiacum* and *H. alvei* lies in their sulfur metabolism. In general, genes involved in cysteine and methionine, selenocompound and sulfur metabolism pathways are downregulated in *B. aurantiacum* in the coculture (Fig. 4C). *B. aurantiacum* is a known producer of aromatic compounds in cheese [24]. The downregulation of the sulfur metabolism and lack of synthesis of VSCs (Fig. 3C) in the presence of *H. alvei* is rather unexpected. To investigate further, we examined oxidative stress-related genes and proteins. Catalases, peroxidases, and a thioredoxin reductase were all downregulated in the coculture, suggesting that the cells were not under oxidative stress (Fig. 4D). Consequently, this also indicates that under iron-limiting conditions, iron starvation could have caused oxidative stress in *B. aurantiacum* monoculture, which was alleviated by *H. alvei* in the coculture.

#### Quorum sensing signaling between *H. alvei* and *B. aurantiacum*

Genes related to QS were upregulated and downregulated in both strains (Figs 2D and 4C). LuxS, an autoinducer-2 (AI-2) synthase protein that is involved in inter-species cross-talk, was upregulated in *H. alvei* in the coculture (1.1 log$_{2}$ fold change) [49]. However, the corresponding receptor was not present in *B. aurantiacum*. Acyl-homoserine lactone (AHL) synthase, which produces autoinducer 1 (AI-1) involved in intra-species cross-talk, was downregulated in *H. alvei* (−1.6 log$_{2}$ fold change). AHL has been shown to affect virulence gene expression, including siderophore synthesis [50]. Siderophore biosynthesis genes were downregulated in *H. alvei* in the coculture (Supplementary Tables S1 and S2). Furthermore, in the coculture, *B. aurantiacum* expresses a dienelactone hydrolase and beta-lactamase (log$_{2}$ fold change > 1), which have been shown to inhibit AI-1 QS signals in other microbes [51, 52]. Volatilome analysis also revealed that 2-hydroxybenzaldehyde, a potential AI-1 inhibiting agent, is present in high quantities in the coculture, and is only present in the *B. aurantiacum* monoculture (Fig. 3C).

## Discussion

**Figure 5:**
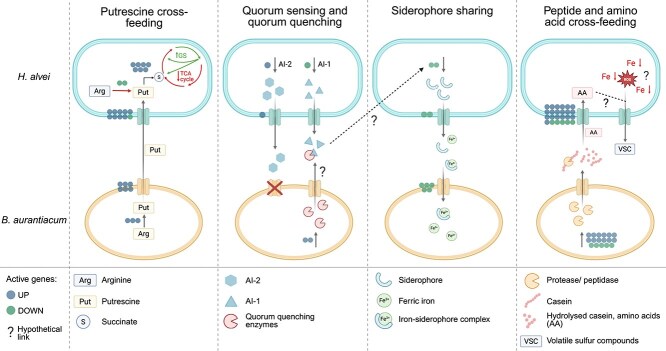
Schematic representation of proposed interactions between *H. alvei* and *B. aurantiacum* in coculture under iron-limitation. The active genes represent upregulated and downregulated genes in coculture compared with monoculture.

In this work, we studied the iron-dependent cross-feeding behavior in the cheese microorganisms *H. alvei* and *B. aurantiacum*. Preliminary experiments showed that *H. alvei* can resist higher iron limitation than *B. aurantiacum*, likely due to its ability to produce siderophores. However, its inability to grow in casein indicates that it has low proteolytic activity compared with *B. aurantiacum*. These initial findings highlight their potential for cross-feeding behavior, where *B. aurantiacum* utilizes the siderophores of *H. alvei*, and *H. alvei* depends on *B. aurantiacum* to access the nitrogen source (Fig. 5).

When grown in the presence of *B. aurantiacum*, *H. alvei* seemed to experience iron starvation, which likely resulted in reduced growth. This was initially observed through a downregulation of the TCA cycle and activation of the GS. The downregulation of the TCA cycle may be linked to reduced iron availability, as [Fe–S] clusters are necessary for the activity of aconitase and succinate dehydrogenase [53]. The activation of the GS as a result of iron deprivation has been reported in microbes from other iron-limiting environments [54–56]. This could be a strategy to direct the flux away from the iron-heavy succinate dehydrogenase. Furthermore, a bifunctional AcnA, which serves as an iron-responsive element under nutrient and oxidative stress, was upregulated. In its apo-form, without the [Fe–S] cluster, AcnA is able to bind its own mRNA to maintain its synthesis due to the essentiality of the TCA cycle [48]. This would explain the upregulation of citrate synthase and the aconitases during the potential iron starvation response induced by the coculture in *H. alvei*. As a result of the disruption of the TCA cycle, as well as iron limitation itself, oxidative phosphorylation was also impaired, as genes in this pathway were mostly downregulated. This suggests that the metabolism had (partially) shifted toward anaerobic respiration, likely due to the dependence of aerobic metabolism on iron, given that we confirm that the cells were not under oxygen-limitation (see Supplementary Fig. S1). This is a possible hypothesis considering that *H. alvei* is a facultative anaerobe [31]. Moreover, the upregulation of formate dehydrogenase N and dimethyl sulfoxide reductase subunits that can form an ETC, further supports this assumption. The same is not observed in *B. aurantiacum*. The combination of not having many changes in the TCA cycle gene expression and slightly upregulated glycolysis/gluconeogenesis and oxidative phosphorylation hints at *B. aurantiacum* experiencing less iron starvation than in the monoculture. This is further supported by the upregulation of high affinity iron transporters in the monoculture, where it is likely that the more extreme iron limitation induced an iron starvation response [57].

The changes in central metabolism were reflected in the profile of volatile compounds. The majority of relevant aromatic compounds contributing to cheese flavor were synthesized in the coculture (Fig. 3B). Among the compounds produced only in the coculture are trimethyl disulfide (sulfurous, cabbage, and garlicky flavor) and 2-tridecanone (“goaty” flavor) (Fig. 3A and C) [58]. It is plausible that the interactions in the coculture can lead to a more well-rounded and improved aromatic profile of cheese. Our multi-omics approach identified these metabolites and a few of the pathways involved in their biosynthesis as important to the coculturing response. Future studies should include an absolute quantification of these compounds to further assess their role in their interactions, but also in the organoleptic characteristics of cheese. One factor that could contribute to sulfur metabolism and the formation of VSCs could be related to oxidative stress. Although oxidative stress is commonly associated with iron excess, instances of iron limitation-induced oxidative stress have also been reported [59, 60]. It remains to be determined whether such a response occurred here, potentially activating sulfur metabolism and resulting in the formation of VSCs.

### Putrescine as a cross-fed metabolite

Polyamines are involved in numerous processes in the cell, such as protecting from oxidative and acid stress; biofilm formation; siderophore synthesis; bacterial swimming motility and swarming; and signaling [61, 62]. Several of these processes depend on the secretion of polyamines into the extracellular environment, which fosters various types of microbial interactions. The necessity of polyamines in widespread metabolic processes suggests that their requirements are shared across different ecosystems. In the coculture of lung pathogens *Acinetobacter baumannii* and *Klebsiella pneumoniae*, putrescine export is increased, with *A. baumannii* showing upregulated 4-aminobutyrate-2-oxoglutarate transaminase involved in putrescine degradation [62]. In the oral cavity, *Streptococcus gordonii* produces ornithine that *Fusobacterium nucleatum* converts into putrescine, which in turn enhances *Porphyromonas gingivalis* biofilm formation [63]. In the gut, acidic conditions induce an acid stress response and energy production in *Escherichia coli* and *Enterococcus faecalis*, sequentially driving putrescine biosynthesis [64]. These examples demonstrate how microbes use putrescine to interact with each other to perform various metabolic functions. We now contribute to the existing body of knowledge regarding polyamine-mediated microbial interactions by highlighting their significance within food systems as well, where similar interactions took place.

We report the upregulation of two putrescine utilization pathways in *H. alvei* when grown in a coculture: PatA and PatD, and the Puu pathway. Both of these pathways lead to the synthesis of gamma-aminobutyrate or GABA, which is subsequently converted to succinate and can enter the TCA cycle [43, 44]. Despite the evidence of the usage of putrescine as an energy source, arginine decarboxylase and agmatinase, involved in arginine degradation to putrescine, are downregulated in *H. alvei* [65]. However, the same genes are upregulated in *B. aurantiacum* in the coculture. In addition, both strains highly express multiple putrescine/spermidine transporters compared with monocultures. These findings indicate that putrescine is being produced by *B. aurantiacum* and potentially utilized by *H. alvei* as a carbon and nitrogen source (Fig. 5). Biogenic amines are generally produced when cells are experiencing acid stress. When *B. aurantiacum* is cultured with *H. alvei*, it could be experiencing mild acid stress (pH 6.6 compared with 6.8 in monoculture, data not shown), similar to the case in the gut microbiome. The large quantity of amino acids from casein proteolysis, together with the slight decrease in pH could induce *B. aurantiacum* to produce putrescine. Generally, biogenic amines can be a concern in surface-ripened cheese because their synthesis is supported by higher rates of proteolysis and extreme conditions for microbial survival. They are implicated in food-borne illnesses, and putrescine-degrading bacteria like *H. alvei* could contribute to a safer product [66, 67].

### Potential disruption of intra-species communication

QS mechanisms have been shown to be present in diverse ecosystems, whether in marine environments, soil, eukaryotic hosts, food industry, various biofilms, and others [68]. The presence of QS signal producers in a range of microbial ecosystems indicates its importance in microbial composition and interactions. However, the disruption of these signals by various quorum quenching (QQ) strategies has also been reported. In the marine environment, although QS affects important biogeochemical processes, QQ molecule-producing marine bacteria belong to a wider phylogenetic diversity than QS-producers [69]. Similarly, in soil microbiomes, a large portion of bacteria produce both QS and QQ signals, namely 10%–20% and 5%–10%, respectively [70]. These findings indicate that microbial interactions can occur through QS and QQ, and could prove to be especially relevant in the cheese surface. Many phenotypes associated with QS are linked to surface colonizing microbes, as QS can influence motility, adhesion, and biofilm formation [71]. Consequently, given the spatial characteristics of cheese, along with the low bioavailability of iron, investigating QS and QQ communication could improve our understanding on microbial interactions beyond the cheese ecosystem.

Virulence factors, including siderophore synthesis, have been reported to be affected by QS signals. The mechanism seems to vary across species. In some bacteria, as in *Burkholderia cepacia* and *Vibrio vulnificus*, siderophore production is reduced at high cell density (thus high QS signals), whereas in *Pseudomonas aeruginosa*, the opposite was reported where knocking out the QS regulator LasR resulted in decreased siderophore production [72–74]. On the cheese surface, AI-2 signals have been shown to be produced by several ripening bacteria, indicating the relevance on the cheese ecosystem [75].

QS was found to be significantly enriched in *H. alvei* but not in *B. aurantiacum* (Fig. 2D). *H. alvei* expressed an AHL synthase, responsible for the synthesis of AI-1, which is used in intra-species cross-talk (Fig. 5). Although AHL synthase was downregulated, it was still expressed in the coculture. In *B. aurantiacum*, dienelactone hydrolase and beta-lactamase were more highly expressed in the coculture, both of which have been implicated in QS inhibition (Fig. 5) [51, 52]. This raises the question of whether these molecules interfered with the intra-species communication in *H. alvei*, resulting in decreased siderophore production. *H. alvei* strains isolated from raw milk were found to synthesize several AHLs, namely $N$-(3-oxo-hexanoyl)-L-homoserine lactone, $N$-(3-oxo-octanoyl)-L-homoserine lactone (3-oxo-C8-HSL), $N$-hexanoyl-L-homoserine lactone, and $N$-octanoyl-L-homoserine lactone [76–78]. In other microbes, inhibition of 3-oxo-C8-HSL has been linked to potential dienelactone hydrolases [79, 80]. However, dedicated experiments need to be carried out to explore this relation. Furthermore, 2-hydroxybenzaldehyde was detected in greater quantities in the coculture and was only present in the *B. aurantiacum* monoculture. In *Pseudomonas aeruginosa*, benzaldehydes have been shown to disrupt AI-1-mediated signaling [81]. It remains to be investigated whether volatile aromatic compounds produced by cheese ripening bacteria, like 2-hydroxybenzaldehyde, could have a role in quorum-sensing communication.

The environment on the cheese surface is complex, with a combination of biotic and abiotic factors affecting the present microbiota. Iron, an element with low bioavailability in cheese, could be one of the determining factors for their survival [10]. As a result, microbial interactions involving iron acquisition have become a strategy to ensure colonization. Some of these modes of interaction may highly depend on the spatial characteristics of the matrix. Although our study underlines the potential for interactions, it does not account for the full complexity of the cheese matrix and metabolite diffusibility within cheese. In the present study, we use two ripening microorganisms, *H. alvei* and *B. aurantiacum* as a simple model to study these interactions in conditions where iron availability can be studied separately from other factors. A mutualistic behavior between these species has already been established in mini model cheeses [23], and the present work furthers our understanding of the mechanisms behind it. We report that under iron-limiting conditions, *B. aurantiacum* alters the central metabolism of *H. alvei*, and favors the production of volatile organic compounds. In addition, *H. alvei* is able to use the putrescine potentially produced by *B. aurantiacum* as a carbon source. These factors contribute to a product that is both safer and has desirable organoleptic properties. Furthermore, with cheese being a host of diverse microbial communities, QS in the context of ripening bacteria interactions has been understudied. Given the strong dependence of cheese-surface bacteria on siderophores for iron acquisition, and the influence of QS on siderophore synthesis across various microbes, a thorough understanding of these signaling mechanisms could improve our understanding of the composition and dynamics of microbial communities on the cheese surface. Similar interactions to the ones we report have been documented in various environments, including several human pathogens, as well as marine and soil ecosystems [62–64, 69, 70, 74]. Despite the differing environments, the fundamental microbial processes are part of a broader strategy employed by microbial communities to survive challenging conditions, including food systems.

## Supplementary Material

Supplementary-Material_wrag100

## Data Availability

The raw transcriptomic data have been deposited to the NCBI repository with the accession number PRJNA1238254. All additional material related to the processing of transcriptomic data, as well as the processed and unfiltered proteomic and metabolomic datasets, are available at the Recherche Data Gouv repository.
