# Supplementary material for: Iron limitation promotes metabolic cross-feeding between cheese ripening bacteria
Source: ISME J. 2026 Apr 24;20(1):wrag100. doi: 10.1093/ismejo/wrag100 (PMC13198182; doi:10.1093/ismejo/wrag100)
Supplement: Supplementary-Material_wrag100 [file supplementary-material_wrag100.zip › Supplementary_Figure_S1_wrag100.pdf]

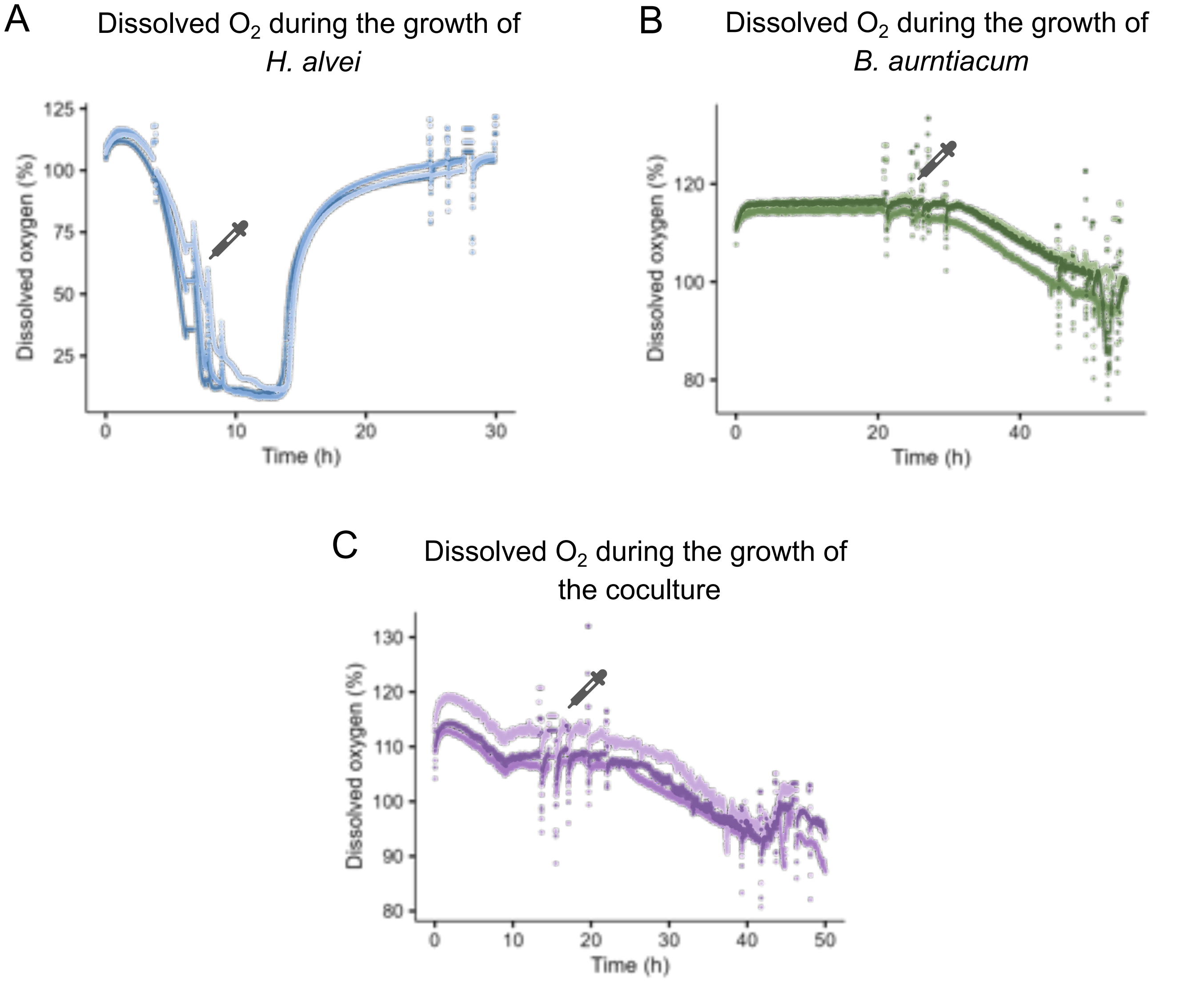

Figure 1: Dissolved oxygen measurements during the growth of: (A) *H. alvei*, (B) *B. aurantiacum* and (C) coculture. Sampling times are indicated by symbol.
